# Targeting the Molecular and Immunologic Features of Leiomyosarcoma

**DOI:** 10.3390/cancers15072099

**Published:** 2023-03-31

**Authors:** Brandon M. Cope, Raymond S. Traweek, Rossana Lazcano, Emily Z. Keung, Alexander J. Lazar, Christina L. Roland, Elise F. Nassif

**Affiliations:** 1 Department of Surgery, Keesler Medical Center, Biloxi, MS 39534, USA; 2 Department of Surgical Oncology, The University of Texas MD Anderson Cancer Center, Houston, TX 77030, USA; 3 Department of Pathology, The University of Texas MD Anderson Cancer Center, Houston, TX 77030, USA; 4 Department of Genomic Medicine, The University of Texas MD Anderson Cancer Center, Houston, TX 77030, USA; 5 UTHealth Houston Graduate School of Biomedical Sciences, The University of Texas MD Anderson Cancer Center, Houston, TX 77030, USA; 6 Department of Sarcoma Medical Oncology, The University of Texas MD Anderson Cancer Center, Houston, TX 77030, USA

**Keywords:** leiomyosarcoma, immune microenvironment, immune-checkpoint blockade, targeted therapy, biomarker

## Abstract

**Simple Summary:**

Immunotherapy has revolutionized cancer care across different cancer types. Unfortunately, leiomyosarcoma does not seem sensitive to the first-generation immune-based therapies. In this review, we present the results of trials of immunotherapy in leiomyosarcoma, emphasizing differences in results between soft-tissue leiomyosarcomas and uterine leiomyosarcomas. Then, we discuss the different molecular subgroups of leiomyosarcomas and how molecular alterations may impact response to immune checkpoint blockade. Based on these molecular descriptions, we propose some future directions to improve response rate of immunotherapy in leiomyosarcoma patients, through (1) better characterization of the immune microenvironment of different leiomyosarcoma molecular subtypes, (2) combination treatments of immunotherapy with therapies targeting specific molecular alterations, (3) new generations of immune-based therapies targeting other components of the immune microenvironment (macrophages).

**Abstract:**

Leiomyosarcoma (LMS) is a rare, aggressive mesenchymal tumor with smooth muscle differentiation. LMS is one of the most common histologic subtypes of soft tissue sarcoma; it most frequently occurs in the extremities, retroperitoneum, or uterus. LMS often demonstrates aggressive tumor biology, with a higher risk of developing distant metastatic disease than most sarcoma histologic types. The prognosis is poor, particularly in patients with uterine disease, and there is a need for the development of more effective therapies. Genetically, LMS is karyotypically complex and characterized by a low tumor mutational burden, with frequent alterations in *TP53*, *RB1*, *PTEN*, and DNA damage response pathways that may contribute to resistance against immune-checkpoint blockade monotherapy. The LMS immune microenvironment is highly infiltrated with tumor-associated macrophages and tumor-infiltrating lymphocytes, which may represent promising biomarkers. This review provides an overview of the clinical and pathologic behavior of both soft tissue and uterine LMS and summarizes the genomic and immune characteristics of these tumors and how they may provide opportunities for the development of biomarker-based immune therapies.

## 1. Introduction

Soft tissue sarcomas (STS) are a heterogeneous group of more than 50 different types of rare mesenchymal tumors [1]. Leiomyosarcoma (LMS) is one of the most common types of STS, representing up to 15% of all newly diagnosed cases [2]. Common locations for tumor development include the extremities, abdomen, the retroperitoneum (usually from large blood vessels [i.e., Inferior vena cava]), and the uterus [3]. Cutaneous leiomyosarcomas—now also termed atypical intradermal smooth muscle neoplasm—are treated distinctly and outside the scope of this review due to their excellent prognosis. LMS of the uterus (uLMS) is the most common type of uterine sarcoma and likely accounts for the single largest site-specific group of LMS [4,5]. Both LMS of the soft tissues (ST-LMS) and uLMS are identified histologically as cells that show distinct features of the smooth muscle lineages or differentiation, expressing various levels of α-smooth muscle actin, desmin, and h-caldesmon, depending on their degree of differentiation [6,7]. However, ST-LMS and uLMS demonstrate distinct biological differences, such as hormone receptor expression (estrogen receptor [ER]/progesterone receptor [PR]) status and malignant potential, which are associated with important clinical differences, staging and prognosis, and treatment patterns [8].

Outcomes for patients with ST-LMS are poor and heterogenous. Overall, the 2- and 5-year disease-free survival for patients with localized disease range from 40 to 80% and 30 to 64%, respectively [9,10]. In patients with advanced disease, the median progression-free survival (PFS) with first-line treatment is 5–12 months [11,12], and the median OS is 12–25 months [11,13,14]. Specific clinicopathologic factors, such as patient age, tumor location, histologic grade, tumor size, tumor depth, and surgical margins, have been shown to affect both local and distant disease control rates in study series [11,12,13,14,15]. 

Although most patients (60%) with uLMS are diagnosed at an early stage, uLMS is associated with a poor prognosis. Recurrence rates vary from 45% to 75%, with a wide range of recurrence sites [16]. The 5-year survival rate of women with metastatic uLMS approaches only 10–15%, with mortality typically seen within 2 years [17] and an estimated median survival of approximately 12 months for those with stage IV disease [18]. 

Thus, new treatments are needed, and a molecular review of both ST-LMS and uLMS may help to better stratify treatment on the basis of molecular subsets. Historically, a “one-size-fits-all” approach has dominated STS therapy to help accrue patients for clinical trials [1]. Thus, much of our knowledge regarding the responsiveness to therapy of individual STS types was a matter of acquired clinical experience rather than a mechanistic biologic understanding [1]. In this review, we use a molecularly driven approach to describe the utility and activity of cytotoxic drugs, targeted agents, and immunotherapies on the basis of their mechanisms of action and target expression in LMS, with an emphasis on the new immunotherapeutic avenues that may be rationally targeted through a better understanding of the molecular subtypes of LMS. First, we focus on conventional treatments, including chemotherapy and targeted therapies, and then we discuss current evidence for the use of immune-checkpoint inhibitors. Finally, we discuss the molecular and immune landscapes of LMS as a rationale for the development of new molecular-specific immune-based therapies. This manuscript outlines potential therapeutic avenues in a subtype specific and molecularly driven approach to improve our current understanding of the immune landscape of LMS and proposes novel immunotherapy approaches for the treatment of LMS. 

## 2. Current Standard of Care Treatment for LMS

LMS should be treated in specialized cancer centers, as it has demonstrated a significant survival benefit [19,20,21]. Pretreatment biopsy is mandatory, with core biopsy as the preferred technique to ensure minimal tissue disruption while simultaneously providing ample tissue for pathologic assessment [3,22].

Surgical resection is the cornerstone treatment for localized LMS, independent of the site of origin [22,23]. The goal of resection is to achieve complete surgical excision with negative microscopic margins. Overall, LMS is among the most chemosensitive of STS types, and neoadjuvant doxorubicin-based chemotherapy is recommended for large (>5 cm) intermediate- to high-grade extremity and uterine LMS [22,23,24] and under investigation for retroperitoneal LMS (NCT04031677). 

The efficacy of doxorubicin-combination regimens and their application in LMS were largely informed by the results of landmark studies and meta-analyses performed in the late 20th and early 21st century in studies of mixed STS types [25,26]. The use of standard chemotherapy agents, namely doxorubicin-based regimens, alone and in combination with other cytotoxic drugs in the first-line setting, offers median OS durations of approximately 12–18 months and overall response rates (ORRs) of 20–30% [27,28] in metastatic or relapsed uLMS and ST-LMS [22,23]. 

Overall, gemcitabine in combination with other agents constitutes a valuable alternative for patients with LMS whose disease has progressed after the failure of prior standard chemotherapy with doxorubicin [22,23,24]. Hensley et al. [29] evaluated the combination of gemcitabine plus docetaxel in 34 patients with both uLMS (29 of 34) and ST-LMS (5 of 34) who had all previously progressed on prior chemotherapy and found an ORR of 53%. Further study of the combination of gemcitabine plus docetaxel versus gemcitabine alone in pretreated LMS validated the synergistic cytotoxicity of this combination in prolonging stable disease, albeit with higher rates of treatment-associated toxicity [30]. 

Trabectedin, a synthetic antineoplastic drug, has been observed to have efficacy in both ST-LMS and uLMS in clinical trials, specifically in patients who have exhausted standard chemotherapy [31,32]. Trabectedin was investigated in a phase III multicenter randomized controlled study in advanced liposarcomas and LMS after failure of doxorubicin: 518 patients were randomized to receive trabectedin (*n* = 345) or dacarbazine (*n* = 173). Trabectedin demonstrated a significant increase in PFS compared to dacarbazine (4.2 months versus 1.5 months, *p* < 0.001) [33]. 

Doxorubicin in combination with trabectedin was evaluated in a recently reported randomized, multicenter, phase III trial (LMS-04) conducted at 20 centers of the French Sarcoma Group [12]. The study included adult patients with metastatic or relapsed unresectable uLMS (*n* = 67) or ST-LMS (*n* = 83) who had not been previously treated with chemotherapy. Patients were assigned to receive either doxorubicin alone or doxorubicin plus trabectedin. The median PFS duration was significantly longer with doxorubicin plus trabectedin versus doxorubicin alone (12.2 months [95% CI, 10.1–15.6] vs. 6.2 months [95% CI, 4.1–7.1]; *p* < 0.0001). 

To evaluate the tolerability of pazopanib, an oral angiogenesis multikinase inhibitor that targets VEGF, PDGF, and c-kit, Sleijfer et al. [34] accrued 142 patients, 42 of whom had high- or intermediate-grade advanced LMS that had received previous therapy. The study observed that 44% of LMS patients were progression-free at 12 weeks after the start of treatment and the median OS duration was ~1 year, with well-tolerated toxicity profiles. Pazopanib was further explored in STS in a phase III study (PALETTE) including 369 patients from 72 institutions worldwide with metastatic or progressive STS, who had undergone at least one regimen containing anthracycline; patients were randomly assigned to pazopanib vs. placebo in a 2:1 ratio, with 44% (*n* = 109) of LMS patients. The trial demonstrated that pazopanib significantly increased the PFS duration by a median of 3 months compared with placebo [35]. 

### 2.1. uLMS-Specific Considerations

Incomplete hysterectomy and morcellation have been associated with reduced overall and intra-abdominal recurrence rates as well as death rates compared to complete hysterectomy [36]. 

Radiation therapy after resection of localized uLMS has been evaluated in several retrospective analyses. A randomized phase III trial conducted by the European Organisation for Research and Treatment of Cancer (EORTC) evaluated the impact of RT on local control whereby 103 patients with stage I and II uLMS disease were randomly assigned to receive adjuvant pelvic radiation therapy (50.4 cGy in 28 fractions over 5 weeks) or observation. No differences were observed in OS or disease-free survival (DFS) [37]. Therefore, adjuvant radiation therapy is generally not recommended [38] in localized uLMS.

Although no trial has formally shown that adjuvant chemotherapy has an OS benefit in LMS, perioperative chemotherapy remains the standard of care for uLMS [39,40]. A prospective randomized phase III study, which was halted because of a lack of recruitment, reported a significantly higher 3-year DFS rate (55% vs. 41%) following the administration of polychemotherapy consisting of doxorubicin/ifosfamide/cisplatin in addition to radiation therapy, but this had no effect on OS [41]. A consecutive prospective one-arm phase II study showed a longer DFS (3-year DFS rate, 57%) in LMS limited to only the uteruses that were treated with gemcitabine plus docetaxel followed by doxorubicin than in historical controls [18]. 

### 2.2. ST-LMS-Specific Considerations

Complete resection is essential for management of patients with localized disease and may be accomplished by limiting dissection to uninvolved tissue planes and resecting the tumor en bloc with wide margins—including, when possible, at least one uninvolved tissue plane circumferentially [42,43]. Incomplete resection has consistently been associated with local and distant ST-LMS recurrences in large series [43,44] and has been demonstrated to be a predictor of poor survival [44,45]. 

Radiation therapy and chemotherapy are important adjuncts in the treatment of ST-LMS. A recent systematic meta-analysis of 16 studies (*n* = 3958) indicated that external beam radiation therapy reduced local recurrence in patients with STS of the extremities, head and neck, or trunk wall. Additionally, local recurrence rates were lower for preoperative radiation therapy than for postoperative radiation therapy [46]. The prospective EORTC-62092 (STRASS) randomized, phase III study, evaluated 266 patients with localized retroperitoneal sarcoma, 20% of whom had ST-LMS; no benefit was demonstrated in terms of RFS or OS in patients treated with neoadjuvant radiation therapy and surgery vs. surgery alone [47]. 

## 3. Emerging Role of Immunotherapy in the Treatment of LMS

Despite efforts to improve the prognosis of uLMS and ST-LMS with chemotherapy combinations, there have been only modest improvements over the past 40 years. LMS treatment represents an important unmet medical need; unfortunately, novel immune-checkpoint blockade (ICB) immunotherapies have been met with disappointing efficacy and long-term outcomes. 

The effect of PD1-targeted ICB (nivolumab) on uLMS was first assessed by Ben-Ami et al. in a phase II study [48]: patients with advanced uLMS who received at least one prior systemic chemotherapy were treated with nivolumab (3 mg/kg IV every 2 weeks) until disease progression or unacceptable toxicity. After inclusion of 12 patients, the median PFS was 1.8 months, with no objective responses; four of the 12 patients died during the 100-day study follow-up period, all as a result of disease progression and the trial was closed for futility. Additional disappointing efficacy in LMS treated by ICBs was observed in SARC028, a multicenter, two-cohort, open-label phase II trial meant to elicit safety and efficacy data on pembrolizumab in multiple different soft tissue types (LMS, liposarcoma, undifferentiated pleomorphic sarcoma [UPS], and synovial sarcoma); no response was observed among the LMS cohort (*n* = 10) [49]. 

Two seminal trials investigated the role of combination ICB with anti-PD(L)1 and anti-CTLA4 in several STS histotypes, including LMS. The phase 2, single-center trial performed at The University of Texas MD Anderson Cancer Center evaluated combination anti-PD-ligand 1 (L1) (durvalumab) and anti-CTLA-4 (tremelimumab) immunotherapy in STS patients who had received at least one previous line of systemic therapy. Patients (*n* = 57) were observed for a median follow-up of 37.2 months; the PFS rate at 12 weeks was 49% across all subtypes and demonstrated the efficacy of these classes in combination for future study [50]. Three LMS patients were included and all of them had progressive disease as their best response. Elsewhere, the Alliance A091401 trial [51] was a multicenter, open-label, randomized, non-comparative trial of heavily pretreated sarcomas (61% of patients had at least three lines of prior chemotherapy) of multiple histological subtypes. Eighty-five patients in the trial, 34% of whom had LMS, received either nivolumab or nivolumab and ipilimumab, followed by nivolumab. An ORR of 15% was observed in the combination arm and 8% in the nivolumab monotherapy arm. The median PFS durations were 2.1 and 4.1 months, respectively, in favor of combination ipilimumab and nivolumab. LMS-specific results were not reported. 

Examinations of LMS treated by ICB in treatment-naïve settings have shown improved efficacy, specifically when used in combination. Chen et al. [52] examined the efficacy and safety of nivolumab plus ipilimumab versus nivolumab alone in individuals with treatment-naïve PD-L1-positive metastatic STS (83 of 150 had ST-LMS). The authors do not report on LMS-specific response but noted a significant difference with a confirmed response rate and PFS in favor of combination treatment versus nivolumab alone. Specific subset analysis based on STS type was not carried out or mentioned by the authors, but they conclude that the findings underline the promise of combined checkpoint inhibition in PD-L1 positive STS, including LMS.

Saerens et al. [53] performed a systematic review of treatment of STS patients with ICB and analyzed 27 articles involving 25 clinical trials and including 1012 patients. LMS was the third most prevalent histologic subtype represented, and uLMS and ST-LMS were individually analyzed. On subgroup analysis, the ORRs using Response Evaluation Criteria in Solid Tumors (RECIST) version 1.1 were 0.06 (95% CI, 0.02–0.18) for uLMS and 0.10 (95% CI, 0.06–0.17) for ST-LMS, which was below the pre-specified cutoff for clinical activity of ORR = 0.15. A second pooled analysis of phase II trials recapitulated these findings; the authors revealed that compared with other selected types of STS, LMS was the least responsive to anti-PD-1/L1 agents, with an ORR of 6.9% when grouping both uLMS and ST-LMS [54]. 

Multiple studies have investigated the efficacy of combining chemotherapy with ICB in LMS. Tan et al. [55] evaluated the efficacy of PD-1 inhibitors combined with doxorubicin and dacarbazine, with and without ICB, in a retrospective single-institution cohort study. In all, 41 patients with metastatic LMS between 2020 and 2022 were evaluated: 21 patients received doxorubicin and dacarbazine alone, while 20 received doxorubicin, dacarbazine, and ICB. The study observed that although the chemotherapy plus ICB group had a higher ORR (30% vs. 4.8%, *p* = 0.04), there were no benefits in terms of disease control, PFS, or OS. No factors, including age, gender, primary tumor site, tumor grade, tumor extent, metastatic site, and previous use of doxorubicin-based regimens, demonstrated predictive value for PFS or OS. A combined phase I/II trial performed by Pollack and colleagues [56] included 37 patients; the most common histologic type was LMS (ST-LMS = 8 and uLMS = 3). UPS, DDLPS, and chondrosarcoma had durable disease regression, with no significant activity in LMS types. In another phase II study of pembrolizumab with doxorubicin in first-line setting, ten patients with LMS were included in the study, four patients demonstrated a partial response, and six demonstrated stable disease as the best response. The median OS for the 30 patients on trial (UPS, DDLPS, and LMS) was 17.0 months [57]. 

The combination of PD-1 inhibitors and tyrosine kinase inhibitors has also been investigated to determine its efficacy in STS [58]. A phase II trial assessed the efficacy of axitinib plus pembrolizumab in multiple STS including four uLMS and two non-uLMS patients: one partial response lasting for more than a year and one minor response for more than 6 months were observed in these six patients; both were noted in a patient with ST-LMS [59]. A retrospective study that included 20 LMS patients among 61 who received PD-1 and tyrosine kinase inhibitors found that LMS patients had the lowest response rate compared with other subtypes, and rapid progression occurred when PD-1 monotherapy was administered [60]. 

Therefore, to date, immunotherapy remains disappointing in LMS, with the suggestion that ST-LMS has better response rates than uLMS (Table 1). Thus, a better understanding of LMS biology may help improve immunotherapy efficacy and the development and application of novel combinations and agents. 

## 4. Molecular Landscape of LMS 

**Figure 1:**
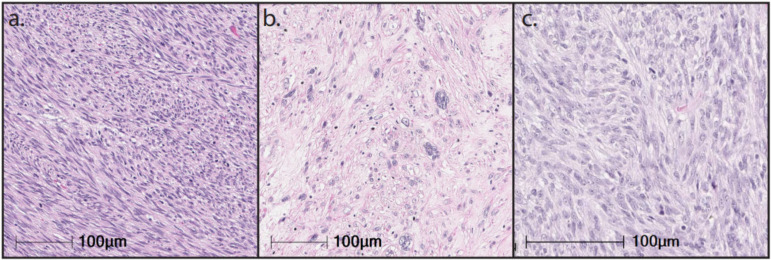
Hematoxylin-and-eosin slides of different leiomyosarcoma subtypes at 20× magnification. (**a**) Well-differentiated soft tissue leiomyosarcoma; (**b**) Dedifferentiated soft tissue leiomyosarcoma; (**c**) Uterine leiomyosarcoma.

LMS encompasses tumors that exhibit a wide range of differentiation with progressive loss of muscle markers extending from well-differentiated to poorly differentiated LMS that resembles UPS. The morphology of LMS and the expression of muscle markers are significant prognostic factors [7]. LMS are genomically complex tumors, with frequent alterations of *TP53* (80%), *RB1* (80%), and *PTEN* (80%) [65,66], and can be subtyped into three genomically distinct clusters: a uLMS subtype and two soft tissue LMS (ST-LMS) subtypes [66,67,68]. The more dedifferentiated ST-LMS subtype is characterized by significantly higher genomic instability more closely resembling UPS and by significantly worse OS. These genomically distinct clusters have significant phenotypic and morphological differences (Figure 1). In this section, we detail the molecular hallmarks of LMS and their potential effect on the tumor immune microenvironment.

### 4.1. Complex Karyotype

The karyotype of LMS is genomically complex, with multiple genes that are frequently altered to varying degrees and no pathognomonic chromosomic alteration. Overall, LMS has a lower tumor mutational burden (median of 2.61 mutations/Mb [69,70]) and fewer somatic copy number alterations (SCNAs) than other complex genomic sarcomas. A higher tumor mutational burden is associated with improved response and survival following ICB [71,72,73]; however, a higher number of SCNAs have been associated with resistance to ICB [74]. 

Both uLMS and ST-LMS demonstrate fewer SCNAs than more immune-sensitive complex sarcomas, such as UPS [66]. SCNAs have been shown to support tumorigenesis and propagate mutagenic processes that drive genomic instability during tumor growth [75]. While some focal SCNAs appear to be preserved across multiple cancer histologies, investigation into LMS has failed to reveal conserved or recurrent copy number alterations [76]. Instead, LMS SCNAs are often highly variable and are characterized by a predominance of deletions [70,77]. Notable among these deletions are genes involved in cell cycle progression and DNA damage repair at rates similar to those found in UPS [76]. A pan-sarcoma molecular analysis of LMS identified deep deletions in *TP53, RB1*, and *CDKN2A* among 9%, 14%, and 8% of LMS [66]. This complex karyotype may inform resistance to ICB because of a paucity of targetable neoantigens and recurrent genetic alterations [78,79,80].

### 4.2. Molecular Subtypes of LMS

ST-LMS and uLMS are molecularly distinct—despite their histologic and karyotypic similarities, they have different methylation and mRNA expression signatures [66]. Initial work to characterize the molecular profile of LMS by Beck et al. used comparative genomic hybridization arrays to cluster LMS into three reproducible subtypes [67]. Extrauterine tumors, which comprised molecular subtypes I and II, tended to have pathways that were enriched for genes encoding muscle differentiation, protein metabolism, and regulation of cellular proliferation. In contrast, uterine tumors, which comprise molecular subtype III, tended to have pathways enriched for genes encoding metal binding, wound response, and ribosomal function and protein synthesis. 

Further work by Guo et al. recapitulated these three molecular subtypes; these authors also identified specific gene signatures enriched within each subtype. Subtype I tumors more frequently overexpressed *LMOD1*, *CALD1*, and *MYOCD*, which are known to be involved with smooth muscle function and differentiation [81]. These findings suggest that subtype I represents a more well-differentiated molecular subtype that is more closely associated with normal smooth muscle function. By contrast, subtype II demonstrated significantly fewer muscle-specific genes than did subtype II and may indicate a more dedifferentiated molecular subtype. Importantly, this dedifferentiated subtype was shown to cluster with undifferentiated pleomorphic sarcomas (UPS). Finally, subtype III was composed almost entirely of uLMS, with 92% of subtype III samples derived from the uterus. Genes enriched in subtype III govern biological processes regulating transcription and metabolic processing and indicate that uLMS comprises a molecularly and clinically distinct cluster from ST-LMS. 

The molecular features of these three subtypes are associated with pathway-specific alterations and may contribute to differences in prognosis. In addition to *MYOCD* amplification, the well-differentiated subtype has been observed to house more frequent mutations in *RB1* than other extrauterine tumors [82]. In addition, up to 84% of dedifferentiated ST-LMS and uLMS are enriched for *PTEN* loss and downstream overexpression of the Akt pathway compared to 44% of *PTEN*-dysregulated subtype II [66]. Conversely, well-differentiated ST-LMS are more hypomethylated than are dedifferentiated ST-LMS, which display a prominent signature of inflammatory cells characterized by NK cell and mast cell infiltration and portends significantly improved RFS survival. Taken together, these data indicate that LMS can be clustered into molecularly distinct and reproducible subtypes that may inform a tailored, molecularly driven therapeutic regimen (Table 2).

### 4.3. TP53

Investigations into frequent molecular alterations in LMS have largely focused on *TP53* mutations, given its established role in oncogenesis among various cancer types [83,84]. *TP53* encodes for p53, a known tumor suppressor that acts as a critical regulator of processes that govern progression through the cell cycle, DNA repair, apoptosis, and cellular senescence [85]. p53 has several metabolic functions that counteract oncogenesis and has been observed to suppress glycolysis and promote oxidative phosphorylation; this, in turn, inhibits glucose metabolism, which has been associated with malignant growth [85]. Metabolic stressors, such as depletion of nutrients or increasingly anaerobic conditions, may also activate p53, preventing tumor progression by driving apoptosis [86]. In this context, loss of p53 function is a common feature in a majority of human cancers, as up to 75% of mutations result in loss of wild-type function [87]. 

Accordingly, up to 80% of LMS demonstrate mutations in *TP53*. Chudasama et al. identified a multitude of chromosomal rearrangements of *TP53* in 49% of their cohort of LMS samples through a variety of mechanisms, including out-of-frame fusions and loss of functional domains [77]. These genetic alterations resulted in *TP53* mutations that clustered most commonly in the DNA binding and tetramerization motifs, indicating substantial heterogeneity in the mutational landscape that drives *TP53* alterations. Notably, alterations in *TP53* appear to be more common in extrauterine LMS, as Hensley et al. identified *TP53* alterations in 71% of ST-LMS versus 56% of uLMS [88]. Among their cohort, the authors identified loss-of-function mutations and homozygous deletions as the most common mechanisms of *TP53* alteration, particularly among uLMS, and noted that *TP53* mutations were more common in LMS than in high-grade non-LMS. Taken together, these data suggest that *TP53* alterations are a common driver of sarcomagenesis in both gynecologic and non-uLMS, although the mechanisms surrounding these mutations are heterogenous. 

Notably, disruptions in *TP53* appear to frequently accompany biallelic inactivation of *RB1.* Chudasama et al. observed biallelic inactivation in over 90% of cases, characterized by a heterogeneity of mechanisms, including protein-damaging microdeletions, inversion, and exon-skipping events [77]. A further genomic analysis identified that while *RB1* is most often deleted, *TP53* may be either deleted or mutated. Using the TCGA, Abeshouse et al. identified shallow deletions in 60% and 78% of *TP53* and *RB1*, respectively [66]. However, mutations were identified in 50% and 15%, respectively, suggesting that while *TP53* and *RB1* are often co-altered in LMS, the exact mechanisms underlying these genetic aberrations may vary. Despite the heterogeneity in molecular mechanisms, these data suggest that concurrent *TP53* and *RB1* inactivation is an oncogenic driver of sarcomagenesis of LMS. 

Characterization of the intratumoral immune environment has revealed associations with several commonly mutated genes, particularly *TP53*. Petitprez et al. developed an immune-based classification system for STS through clustering of tumors based on transcriptomic immune signatures. In doing so, the authors observed a distinct cluster of tumors that was characterized by a higher density of immune infiltrate [89]. Mutations in *TP53* were the most frequently observed genetic aberration among immune-rich groups, occurring in 35.2% of samples. On the basis of these findings, there may be an association between specific genetic alterations and the immune microenvironment, which may have substantial implications for response to ICB. 

### 4.4. RB1

Homozygous deletions in *RB1* are among the most frequent genetic alterations identified in LMS, occurring in as many as 80% of samples [66,90]. *RB1* encodes retinoblastoma 1 (Rb1), which is a well-described regulator of the cell cycle. Under physiologic conditions, mitogenic signals activate CDK4/6, which in turn complexes with D-type cyclins. These kinases phosphorylate and inactivate RB1, which then de-represses the E2F transcription factor and allows progression of the cell cycle [91]. Deletions in RB1, therefore, result in unchecked cellular proliferation and have been implicated in oncogenesis across a variety of human cancers. In addition, there has been emerging evidence of non-canonical roles of RB1 in tumor metabolism, the tumor microenvironment, and epigenetics; these pathways remain poorly understood, and the clinical implications are yet to be determined [91,92,93].

Fusion events affecting *RB1* are less common than deletions but may represent a critical mechanism for driving tumorigenesis, particularly in uLMS. Choi et al. observed *RB1* fusions in 30% of sampled uLMS by RNA-seq resulting in truncation of the RB1 protein and ultimately loss of function [94]. By contrast, fusion events affecting other known LMS driver genes were less commonly observed (8% *TP53* and 8% *ATRX*). In addition, the rate of fusion events among ST-LMS appears less frequent. Liu et al. observed only one fusion event among their cohort of 20 ST-LMS patients, which is significantly lower than the 88% verified in their cohort of DDLPS patients. Fusion transcripts may be a source of neoantigen formation; in an in vitro analysis, fusion-associated neoantigens elicited cytotoxic CD8+ T-cell responses, even among cancers with low tumor mutational burden [95]. Thus, *RB1*-associated fusion events may represent a promising immune target for tailored immunotherapy, particularly in uLMS. 

There is a growing body of evidence suggesting that the genetic aberrations governing cell cycle progression contribute to shaping tumor immunogenicity and the immune microenvironment [96,97]. In particular, the RB1-CDK4/6 pathway has gained interest, given its role in T-cell function. CDK4/6 inhibition has been observed to result in greater IL-2 production, which augments antitumor immunity by enhancing T-cell activation and increasing intratumoral T-cell infiltration [98]. To this end, preclinical studies and case reports have demonstrated promising results of CDK4/6 inhibition in overcoming PD-1 inhibitor resistance in several solid tumors [99,100,101], providing a rationale for the exploration of combination therapy in prospective clinical trials (NCT04438824, NCT05139082) (Table 3). 

RB1 dysregulation may also represent a mechanism of resistance in patients treated with ICB. Previous studies demonstrated that loss of RB1 is associated with decreased leukocyte recruitment, resulting in tumor immune evasion across a variety of cancer histologies [102,103]. Clinically, this immunologic exclusion may contribute to ICB resistance. An in situ analysis of melanoma samples that were resistant to ICB identified tumors that were characterized by immune evasion of intratumoral exclusion of T cells [104]. These resistant tumors demonstrated post-treatment enrichment of *CDK4*; subsequent in vivo CDK4/6 inhibition potentiated anti-PD-1 therapy in these treatment-resistant cells, resulting in decreased tumor growth and greater T-cell infiltration than anti-PD-1 therapy alone. These data indicate important crosstalk between cell cycle regulators and the immune system and may represent a promising avenue for targeted cancer therapeutics. 

### 4.5. PTEN Deletion and PI3k/AKt/mOR Pathway

Multi-platform profiling has identified loss of *PTEN* expression in up to 32% and 38% of ST and uLMS, respectively [105]. *PTEN* encodes a dual-phosphatase protein that inhibits AKT signaling, which activates the downstream mTOR pathway that inhibits cellular proliferation, cell growth, and metabolism and promotes susceptibility to apoptosis [106,107]. As such, PTEN loss results in increased levels of PIP3 and an upregulated PI3k-Akt pathway that stimulates unchecked cell growth [108]. The most frequent mechanism of *PTEN* dysregulation is loss of chromosome 10q, which occurs in as many as 59% of LMS tumors in some studies; its loss may be correlated with more aggressive behavior and the development of metastatic disease [109]. 

The frequency of *PTEN* alterations appears to vary by anatomic location, disease stage, and analysis method. Zhang et al. used molecular analysis by PCR to identify *PTEN* deletions among 32% of primary uLMS samples [110]. These findings were recapitulated with a subsequent FISH analysis, and patients with *PTEN* loss were observed to have significantly worse OS than those who did not. Schaefer et al. observed PTEN protein loss by IHC in 41% and 31% of primary and non-primary uLMS, respectively [90], and 47% and 55% of primary and non-primary ST-LMS. Conversely, Chudasama et al. observed genomic deleterious aberrations in *PTEN* among 57% of LMS samples [77], but the authors did not specify the distribution of anatomic location or disease stage. These data suggest that aberrations in *PTEN* are a common, although non-unifying, feature of LMS and may represent a biomarker of aggressive tumor biology. 

Bi-allelic loss of *PTEN* in LMS has become increasingly associated with resistance to ICB. In vitro experimentation suggests that this is a result of decreased neoantigen formation and subsequent reduction in T-cell immunoreactivity [62]. Therapeutic resistance may be a result of transcriptomic changes associated with biallelic *PTEN* loss, as treatment-resistant tumors have been observed to have significantly lower expression of genes associated with immunologically reactive cytokines, including *PDCD1*, *CD8A*, *IFNG*, and *GZMA*. In this context, a reduction in immunologic reactivity as a result of *PTEN* loss may also contribute to tumoral immune evasion and exclusion. Preclinical models using melanoma cell lines have demonstrated decreased T-cell infiltration in *PTEN*-silenced tumors and show that loss of PTEN upregulates inhibitory cytokines CCL2 and VEGF, both of which have been implicated in tumor immune evasion [111,112,113]. 

PTEN loss has been observed to be correlated with a significant reduction in PD-1+ cell infiltration within uLMS, particularly among metastatic lesions [62]. Treatment-resistant metastatic uLMS lesions have significant reductions in PD-1+ staining following anti-PD-1 therapy, with PD-1 positivity in less than <1% of overall cellularity. As aforementioned, these treatment-resistant lesions are genomically characterized by biallelic *PTEN* loss and upregulation of *VEGFA*; taken together, these data indicate that *PTEN* loss promotes an immunologically excluded tumor microenvironment through upregulation of inhibitory cytokines and downregulation of PD-1, suggesting that *PTEN* significantly mediates acquired resistance to ICB monotherapy [62]. 

### 4.6. DNA Damage Response

The use of next-generation sequencing has provided significant insight into the DNA damage repair signatures that are becoming increasingly identified among LMS [114]. These alterations in DNA damage repair mechanisms are characteristic genomic features that typically occur as a result of deficiencies in homologous recombination repair [115]. Chudasama et al. used whole-exome sequencing to demonstrate hallmarks of BRCAness in 90% of sequenced LMS, including deletions or other structural rearrangements in the genes governing homologous recombination repair [77]. Notable among these genes were *PTEN* in 57% of cases, *BRCA2* in 53%, *ATM* in 22%, and *BRCA1* in 10%. There seems to be a higher proportion of alterations in the DNA damage response genes in the uterine LMS than in soft-tissue LMS [116]. Further, alterations in these genes have been associated with a lower OS in patients with LMS [116]. There has been particular interest in exploring the therapeutic benefit of a given tumor’s BRCAness, as this particular phenotype has been associated with increased sensitivity to PARP inhibitors [117]. 

In addition to PARP inhibitor sensitivity, genomic instability resulting from DNA damage response deficiencies appears to modulate the tumor immune microenvironment. The presence of ongoing DNA damage and deficient DNA damage repair mechanisms has been observed to foster inflammatory signaling that results in intratumoral influx of immunosuppressive cells, particularly myeloid-derived suppressor cells and tumor-associated macrophages (TAMs) [118]. As a result of this immunosuppressive environment, DNA damage continues in the tumor microenvironment via persistent free radical release, ultimately facilitating cancer progression [119,120]. The administration of PARP inhibitors has been observed to produce a more robust infiltration of cytotoxic CD8+ T cells. This has been hypothesized to be a result of cytosolic DNA formation caused by double-strand DNA breaks generated by PARP inhibition, which results in increased secretion of proinflammatory cytokines IL-2, TNFα, and IFNγ, leading to subsequent CD8+ T-cell activation [121,122]. In this context, PARP inhibition may facilitate a more susceptible immune microenvironment, providing a rationale for combination therapy with immune-checkpoint inhibition. 

### 4.7. Whole-Genome Doubling

In addition to gene fusion events, there are emerging data suggesting that whole-genome duplication contributes to LMS progression. Chudasama et al. identified evidence of whole-genome doubling in over 50% of LMS cases [77]. However, the authors note that mutant TP53 and RB1 were detectable irrespective of ploidy, suggesting that tetraploidization preceded the loss of wild-type TP53 and RB1. In addition, whole-genome doubling was more commonly identified among metastatic LMS, suggesting that tetraploidization is a progression event resulting in perturbed tumor suppressor function, ultimately driving gross genomic instability and accelerating tumor evolution. To date, the impact of whole-genome doubling on the immune microenvironment is unknown.

### 4.8. Alternative Lengthening of Telomeres

Alternative lengthening of telomeres (ALT) is a telomerase-independent mechanism by which cancerous cells of typically mesenchymal origin achieve replicative immortality [123,124]. Critical to ALT is the formation of telomeric DNA via homologous recombination, and ALT has been associated with mutations in *ATRX*, a chromatin remodeling factor that regulates histone H3.3 in telomeric and pericentromeric regions of DNA [125,126]. Phenotypically, ALT cells are characterized by the presence of extrachromosomal telomeric sequences that predominantly form single- and double-stranded telomeric circles, termed c-circles and t-circles, respectively [127,128]. Chudasama et al. identified *ATRX* alterations and the presence of c-circles in 78% of LMS samples, supporting previous observations of ALT in mesenchymal tumors [77]. The presence of an ALT phenotype has been associated with poor OS in LMS and has been hypothesized to induce chromosomal instability that contributes to the development of therapeutic resistance [129,130]. While the exact mechanisms underlying the recombination event remain poorly understood, there has been increasing interest in identifying the clinical utility of the ALT phenotype. 

Investigation into the targetability of the ALT phenotype has yielded promising results in the preclinical setting. In particular, the ALT phenotype may confer susceptibility to targeted therapeutics that restore proper cell cycle regulation. Flynn et al. examined multiple ALT-positive cell lines, including osteosarcoma, and observed the creation of recombinogenic nucleoprotein structures associated with persistent activation of replication protein A [131]. When ALT-positive cells were cultured with an inhibitor of ATR, a critical regulation of recombination processes driven by replication protein A, there was significantly more chromosomal fragmentation, with subsequent apoptosis, in ALT-positive cell lines than in controls. 

There has been recent interest in restoring p53 function in ALT-positive cancer cells. Macha et al. examined a small cohort of ALT-positive cancer cells across multiple histologies, including rhabdomyosarcoma and osteosarcoma. The authors observed genomic alterations in *TP53* in 69% of samples by whole-exome sequencing [132]. On the basis of these findings, the authors cultured ALT-positive cell lines with APR-246, which has been observed to covalently bind to and stabilize the core domain of p53, effectively restoring p53 function [133,134], and identified increased cellular apoptosis in ALT-positive cells compared to telomerase-positive controls. Taken together, these data suggest that the ALT phenotype represents a usable biomarker of sensitivity to various targeted therapeutics.

## 5. Immune Landscape of LMS

The immune landscape of both ST-LMS and uLMS is characterized by a lower density of intratumoral immune cell infiltration than other sarcoma histologies with complex karyotypes [66]. However, molecular subtyping of LMS has provided the framework for more rigorous investigation into the immune microenvironment [67]. The presence of tertiary lymphoid structures and PD-L1 has garnered increasing interest, given their association with response to ICB, particularly in UPS and dedifferentiated liposarcoma [89,135]. This section thus outlines the recurring characteristics of the LMS immune microenvironment and details the burgeoning therapeutic potential of these molecular features. 

### 5.1. Tertiary Lymphoid Structures

The presence of tertiary lymphoid structures (TLS) in the tumor immune microenvironment has gained significant interest because of its association with response to ICB. Using transcriptomics data from The Cancer Genome Atlas (TCGA), Petitprez et al. observed that the immune microenvironment of sarcomas could be subtyped in to five distinct immune clusters, named sarcoma immune classes (SIC) ranging from SIC A (immune low) to SIC E (immune high). The SIC was further validated as a predictive biomarker of survival with ICB in the SARC028 trial, whereby patients with more immune-high SICs (E and D) had improved PFS and ORRs compared to immune-low SICs (A and B). The immune-rich SIC E tumors were characterized by high expression of the B lineage signature [89]. Notably, 82% of SIC E tumors were positive for intratumoral TLS by IHC, suggesting that TLS is a marker of effective antitumor immunity. 

On the basis of these data, there has been increasing interest in prospectively examining PD1 blockade in TLS-positive STS. Italiano et al. performed a multi-institutional phase II clinical trial examining pembrolizumab in TLS-positive STS across multiple histologic subtypes, including LMS. The authors observed a median PFS duration of 4.1 months and median OS of 18.3 months in TLS-positive tumors, which was longer than the median PFS of 1.4 months and median OS of 14.3 months in TLS-negative tumors [136]. A subsequent analysis using spatial deconvolution identified significantly increased enrichment of regulatory T cells among TLS-positive non-responders, suggesting that additional elements of the intratumoral immune infiltrate further modulate response to ICB. However, the authors note that TLS is infrequent in LMS, identifying TLS-positive tumors in 12.2% of ST-LMS samples. Petitprez et al. corroborated this observation, noting that most LMS clustered to the immune-low SIC A and B and demonstrated an overall paucity of intratumoral TLS [89]. As such, the utility of using TLS as a biomarker of response to ICB in LMS is crucial in screening for the +/−10% of patients who are more likely to respond to ICB monotherapy and those who may benefit from combinatorial approaches. Since dedifferentiated ST-LMS more closely resemble UPS in clinical behavior, pathology, and molecular markers, these TLS-positive LMS tumors may overlap with this LMS subtype, which requires further validation.

### 5.2. PD-L1 

PD-L1 expression is a controversial biomarker of response across tumor types, as its prognostic and predictive value is often imperfect, depends on the type of ICB used, and the method of evaluation of the biomarker.

Early studies exploring PD-L1 expression in LMS were limited by small cohorts and inconsistent protein expression. D’Angelo et al. used IHC to stain for both tumor cell– and macrophage-associated PD-L1 expression in a cohort of LMS samples. The authors identified macrophage-associated PD-L1 expression in 25% of LMS samples; however, the sample size was small, and the authors were unable to identify tumor PD-L1 expression [137]. Similarly, other studies have found PD-L1 protein expression to be around 30%, with significantly higher proportion of PD-L1 positive in high-grade LMS compared with low grade LMS, indicating once again that higher grade, more dedifferentiated LMS may have a more inflammatory, immune infiltrated tumor microenvironment [138]. Another study by Kim et al., on the other hand, identified PD-L1 expression in 70% of the LMS samples by IHC. However, the authors noted that there was a tendency toward a higher tumor stage among LMS patients; as such, the increase in PD-L1 expression may reflect more advanced tumor biology as a result of more severe disease [139]. 

In contrast to protein expression, the presence of PD-L1 mRNA appears significantly more robust in the LMS immune microenvironment. The results of a RNA-seq analysis suggested that LMS has one of the highest expression levels of PD-L1 of sarcoma histologic subtypes; PD-L1-high tumors were observed in as many as 58% of LMS samples [140]. PD-L1 expression was also significantly associated with lower metastasis-free survival on Cox regression analysis, regardless of other clinicopathologic features. 

### 5.3. Tumor-Infiltrating Lymphocytes 

Beyond B cells and TLS, the presence of tumor-infiltrating lymphocytes (TILs) has gained increasing interest because of its association with the presence of immune-checkpoint markers. In particular, TILs have been associated with TIM-3 and LAG-3, both of which contribute to T-cell exhaustion and may facilitate resistance to anti-PD1/PDL1 [141,142]. Dancsok et al. examined TIL profiles among a large cohort of patients with STS. When categorizing STS according to karyotype, the authors observed that complex-karyotype tumors, including LMS, contained a significantly higher density of TILs than simple-karyotype or chromosomal translocation-associated sarcomas [143]. LMS was specifically characterized by higher densities of CD8+ cytotoxic T cells, CD4+ helper T cells, FOXP3+ T-regulatory cells, and natural killer cells. Despite these findings, intratumoral expression of PD-L1 was infrequent, as the authors observed PD-L1 staining by IHC in 22% of samples. However, a higher TIL score was not correlated with the expression of other immune-checkpoint markers, as the authors observed 58% and 74% of the LMS samples expressing LAG-3 and TIM-3, respectively. In addition, a higher density of TILs was associated with improved OS and PFS in complex-karyotype tumors. 

Recent data suggest that LAG-3 represents an emerging target for ICB, as LAG-3 inhibition has yielded promising results in melanoma. LAG-3 is a cell-surface protein that is typically expressed on activated CD4+ and CD8+ T-cells. When present, LAG-3 binds to MHC class II with high affinity and acts as a competitive inhibitor to CD4, negatively regulating T-cell proliferation and effector T-cell function [144,145]. LAG-3 is often co-expressed with PD-1 on TILs, which act as inhibitory immune checkpoints and have been thought to contribute to tumor-mediated T-cell exhaustion [146,147]. Importantly, recent trials in melanoma in the neoadjuvant and first-line setting demonstrated the superiority of the combination of anti-LAG3 and anti-PD1 therapy compared to anti-PD1 alone [148,149]. High LAG-3 expression has been associated with higher grade, more advanced disease, and significantly worse disease-free survival in patients with STS [150]. Taken together, these findings indicate that LAG-3 is an actionable target, with demonstrated efficacy in both resectable and non-resectable cancers; the clinical utility of LAG-3 inhibition in LMS is yet to be determined. 

In addition, recent preclinical studies have explored the co-expression of PD-L1 and IDO1. Iwasaki et al. cultured LMS-derived cell lines with a JAK2 inhibitor, which targets the pathway regulating IFN-γ-mediated expression of downstream *PD-L1* and *IDO1* [151]. The authors identified significantly downregulated expression of *PD-L1* and *IDO1* mRNA, which was confirmed at the protein level via Western blot analysis. Taken together, these data support an emerging role for combination immunotherapy in LMS patients tailored to the immune microenvironment; accordingly, a clinical trial examining IDO1 inhibitor BMS-986205 in combination with anti-PD-1 therapy in advanced solid tumors was recently completed, and the results are pending (NCT03792750) [152]. 

Both OX40 and TIGIT have emerged as potential candidates for immunologic phenotyping of sarcoma patients. OX40 is a stimulatory ligand that is expressed on activated CD4+ and CD8+ T cells that bind to the TNF receptor family and has been associated with improved survival and reduced T-cell exhaustion [153,154]. Melake et al. observed that the *OX40* transcript was significantly higher in immunologically hot LMS than in immunologically cold LMS [155]. In contrast to OX40, the presence of intratumoral TIGIT may represent an exhaustive phenotype, particularly among NK cells, and be correlated with more aggressive tumor biology. TIGIT is an inhibitory receptor that is upregulated on both NK and T cells in response to chronic antigen stimulation and has been associated with exhaustion and limited antitumor immunity [156,157]. A higher ratio of intratumoral to peripheral TIGIT-expressing NK cells has been associated with disease recurrence in STS, and preclinical experimentation suggests that TIGIT blockade prevents NK cell exhaustion and potentiates response to anti-PD-L1 therapy in STS [158,159]. 

### 5.4. Macrophages

There is an increasing body of evidence suggesting that macrophages play a significant role in the development and progression of epithelial malignancies [160,161]. Macrophages are often recruited to sites of disease and contribute to progression of cancer through the release of growth factors and cytokines that ultimately support angiogenesis [162]. The presence of TAMs has thus been associated with a worse prognosis in various cancer histologies, including kidney, bladder, esophageal, and breast cancer [163,164,165,166]. Initial studies to characterize the immune microenvironment in LMS have corroborated these findings; dense infiltration of CD68+ TAMs and CD163+ TAMs was identified in 26% and 44% of LMS samples, respectively. Notably, both the presence and density of TAMs were associated with a worse prognosis in both uLMS and ST-LMS [167]. On Kaplan–Meier survival analysis, there was significantly improved OS in both uLMS and ST-LMS samples with sparse density of CD68+ and CD163+ TAMs, with an estimated 5-year disease-specific survival of 100% in sparse CD163+ ST-LMS compared to 40% and 70% in dense and moderate CD163+ ST-LMS tumors, respectively. 

Checkpoint-associated proteins may modulate the behavior of TAMs and play an important role in immunologic evasion. In particular, CD47 has gained interest because of its frequent expression in several sarcoma histologic subtypes. CD47 is a transmembrane protein that interacts with SIRPα expressed on macrophages and dendritic cells to prevent phagocytosis [168]. Among STS, CD47 expression appears bimodal, with the highest protein expression level observed in chordoma, angiosarcoma, and pleomorphic liposarcoma; CD47-expressing tumors are associated with worse OS than are CD47-negative tumors [169]. Anti-CD47 therapy has yielded promising results in early-phase clinical trials among patients with both solid and liquid tumors [170,171], and in vitro experimentation suggests that anti-CD47 therapy induces the expression of proinflammatory cytokines IL2, TNFα, and IFNγ in STS [172]. As such, anti-CD47 therapy may represent a promising therapeutic option for LMS patients, particularly given the macrophage-rich tumor microenvironment, and warrants further investigation.

### 5.5. Association of Molecular Subtypes with Immune Microenvironment

The characterization of LMS into distinct molecular subtypes has provided additional insights into the clinical significance of the immune microenvironment. In addition to recapitulating the molecular subtypes, as described in previous studies, Anderson et al. identified a higher density of intratumoral infiltrating immune cells largely dominated by M2 macrophages in their cohort of molecular dedifferentiated ST-LMS than in other molecular LMS subtypes [65]. 

## 6. Conclusions

**Figure 2:**
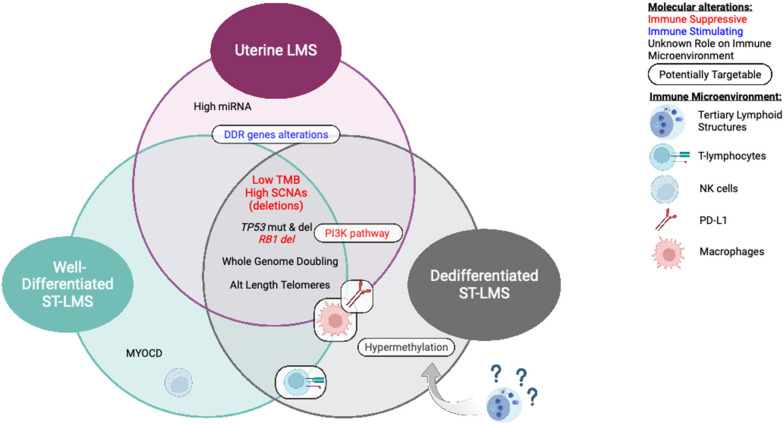
Molecular alterations and immune microenvironment associated with molecular subtypes of LMS.

Although investigations of current ICB have been disappointing in LMS to date, better characterization of molecular and immune subtypes will provide a rationale for new immune-based therapeutic investigations (Figure 2). This review summarized current therapeutic strategies, our current limitations from a multidisciplinary evaluation, and several potential future avenues to investigate, but many others are still under preclinical and clinical investigation.

For the field of immunotherapy to move forward in LMS, we need a better characterization of the immune microenvironment of the three major molecular subgroups of LMS (uLMS, well-differentiated low-grade ST-LMS, and dedifferentiated high-grade LMS). Overall, the tumor microenvironment of uLMS is likely to be very different from other ST-LMS, as the uterus is an organ that physiologically has to be more tolerogenic to host pregnancies. The field lacks data comparing the immune microenvironments of these very different type of tumors.

Clinically, the development of immunotherapy has lumped all LMS together in trials of ICB, which has pushed investigators to believe that all molecular subtypes of LMS are immune-resistant. However, when looking attentively at the data, we have signs that ST-LMS has a higher response rate (10%) to ICB than uLMS (6%), although no formal comparison can be made. To move the field forward, we need to separate high-grade dedifferentiated ST-LMS, low-grade well-differentiated ST-LMS, and uLMS in clinical trials. High-grade dedifferentiated ST-LMS, which has a more immune-inflamed tumor microenvironment, is more likely to benefit from combinations of ICB with PI3K inhibitors, as they more frequently harbor PI3K/Akt pathway immune-suppressive activation. Within this subgroup of dedifferentiated LMS, some of these tumors resembling UPS may be TLS-positive and benefit from a combination of ICB such as anti-PD1 and CTLA4, or more novel ICBs such as anti-LAG3. However, it remains to be determined which molecular subgroup of LMS I more likely to be TLS-positive. Some vascular ST-LMS may benefit from a combination of ICB with antiangiogenic therapies. Another avenue to explore is the potential role for combination with epigenetic drugs, as there is a strong rationale for synergism between epigenetic drugs and ICB, and that LMS subtypes have different methylation signatures. uLMS may benefit from combinations of ICB with DNA damage response pathway targeting drugs. All subtypes of LMS are likely to benefit from the new macrophage-targeting drugs that are currently being developed in phase 1 trials, with dedifferentiated LMS being more likely responsive as it is more heavily infiltrated with macrophages.

## Figures and Tables

**Table 1:** Use of immunotherapy checkpoint blockade in the treatment of leiomyosarcoma.

Type of LMS	Molecule Tested	Target	Design	ORR	DCR (ORR + SD)	PFS and OS Duration, Median (Range)	Reference
uLMS	Nivolumab	Anti-PD1	Phase II	0%	8.3%	PFS: 1.8 months (0.8-unknown) OS: not met; 4 of 12 died during the 100-day study follow-up period because of progression	[48]
	Pembrolizumab	Anti-PD1	Retrospective case study	100%	100%	NA	[61]
	Pembrolizumab	Anti-PD1	Retrospective case study	100%	100%	NA	[62]
ST-LMS	Pembrolizumab	Anti-PD1	Phase II	0%	60%	* PFS: 15 weeks (8–21) * OS: 49 weeks (24–73)	[49]
	Ipilimumab plus nivolumab	Anti-PD1 and anti-CTLA-4	Retrospective	13%	NR	PFS: 4.1 months (3.2–4.5) OS: 12.2 months (6.1–13.7)	[52]
	Nivolumab	Anti-PD1	Retrospective case study	100%	100%	NA	[63]
Both	Iplimumab plus nivolumab	Anti-PD1 and anti-CTLA-4	Phase II	14.3%	NR	PFS: 4.1 months (2.6–4.7) OS: 14.3 months (9.6-nr)	[51]
	Pembro plus axitinib	Anti-PD1 and VEGF inhibitor	Phase II	* 25%	53.1%	* PFS: 4.7 months (3–9.4) * OS: 18.7 months (12-nr)	[59]
	Pembrolizumab plus doxorubicin	Anti-PD1	Phase II	40%	100%	* PFS: 5.7 months (4.1–8.9) * OS: 17.0 months (9.9-nr)	[57]
	Pembrolizumab plus doxorubicin	Anti-PD1	Phase I/II	20%	50%	* PFS: 8.1 months (7.6–10.8) * OS: 27.6 months (18.7-nr)	[56]
	Durvalumab plus tremelimumab	Anti-PDL1 and anti-CTLA-4	Phase II				[50]
	Avelumab plus trabectedin	Anti-PDL1	Phase I/II	* 13%	* 43%	* PFS: 8.3 months Study was halted as it did not meet the primary objective response rate endpoint	[64]
	PD-1 inhibitors (pembrolizumab/toripalimab/sintilimab) plus standard chemotherapy	Anti-PD1	Retrospective cohort study	17.1%	73.2%	PFS: 8.8 months (4.57–13.0) OS: not reached; no difference in OS was observed for chemotherapy alone and chemotherapy plus PD-1	[55]
	PD-1/PDL1 inhibitors plus TKI	Anti-PD1/PDL1 and VEGF inhibitor	Retrospective cohort study	0%	50%	* PFS: 11.74 months	[60]

LMS, leiomyosarcoma; ORR, overall response rate; DCR, disease control rate; PFS, progression-free survival; OS, overall survival; PD-1/PDL1, programmed death-1/programmed death ligand 1; uLMS, uterine leiomyosarcoma; ST-LMS, soft tissue leiomyosarcoma; CTLA, cytotoxic T-lymphocyte associated protein; VEGF, vascular endothelial growth factor; TKI, tyrosine kinase inhibitor; NR, not reported. * Rates of all STS subtypes in the study.

**Table 2:** Characterization of LMS as defined by three major molecular subtypes.

LMS Subtype	Prognosis	Mutational Burden	Cellular Lineage	Molecular Characteristics	Gene Expression	Immune Microenvironment
Well-differentiated ST-LMS	Better	Low	Vascular and digestive smooth muscle	*MYOCD* amplification and upregulated muscle-associated transcripts *TP53* mutations	Enriched: PDGFRA, LRRC15, IGF1R	Inflammatory NK cell signature
Dedifferentiated ST-LMS	Poor		Vascular smooth muscle	*DMD* deletion Reduced markers of muscle differentiation *PTEN* loss, overexpression of Akt pathway *RB1* and *TP53* mutations	Enriched: *ACTA1*, *SYNM*, *LMO1*	Higher immune infiltration overall, dominated by M2-Macrophages Higher leukocyte count overexpression
uLMS	Poor		Gynecologic (uterine, vaginal, fallopian tube) smooth muscle	*DMD* expression inhibition *PTEN* loss, overexpression of Akt pathway *TP53* mutations *RB1* fusion and loss of function	Enriched: ESR1, PGR, EMX2	M2 macrophage-dominant

**Table 3:** List of active and recruiting trials examining immune-checkpoint blockade in leiomyosarcoma.

	Primary Intervention	Secondary Intervention	NCT	Tumor Type	Phase	Status
Immunotherapy						
	Durvalumab	Tremelimumab	NCT02815995	Advanced/metastatic STS	II	Active, not recruiting
	Nivolumab	Ipilimumab	NCT02428192	uLMS	II	Active, not recruiting
	Itacitinib		NCT03670069	Advanced/metastatic STS	I	Recruiting
	LMP1/2 CTLs		NCT01956084	ST-LMS	I	Active, not recruiting
	Nivolumab		NCT03241745	Uterine sarcoma	II	Active, not recruiting
	Tabelecleucel		NCT04554914	ST-LMS	II	Recruiting
	Talimogene laherparepvec		NCT02923778	ST-LMS	II	Recruiting
Immunotherapy + anti-angiogenics						
	Durvalumab	Olaparib, cediranib	NCT03851614	ST-LMS	II	Active, not recruiting
	Nivolumab	Sunitinib, epirubicin	NCT03277924	Advanced/metastatic STS	I/II	Recruiting
	PD-1 inhibition	BA3011	NCT03425279	ST-LMS	I/II	Recruiting
	Pembrolizumab	Eribulin	NCT03899805	ST-LMS	II	Active, not recruiting
Immunotherapy + chemotherapy						
	APX005M	Doxorubicin	NCT03719430	Advanced/metastatic STS	II	Recruiting
	Cabozantinib	Temozolomide	NCT04200443	ST-LMS, uLMS	II	Recruiting
	Nivolumab	Rucaparib	NCT04624178	ST-LMS	II	Active, not recruiting
	Nivolumab	BO-112	NCT04420975	ST-LMS	I	Active, not recruiting
	OR2805	Cemiplimab, docetaxel	NCT05094804	Advanced/metastatic STS	I/II	Recruiting
	Pembrolizumab	Gemcitabine	NCT03123276	ST-LMS	I/II	Active, not recruiting
	Pembrolizumab	Cyclophosphamide	NCT02406781	ST-LMS	II	Active, not recruiting
	Ribociclib	Doxorubicin	NCT03009201	Advanced/metastatic STS	I	Active, not recruiting
	TTI-621	Doxorubicin	NCT04996004	ST-LMS	II	Recruiting

Abbreviations: STS, soft tissue sarcoma; uLMS, uterine leiomyosarcoma; ST-LMS, soft tissue leiomyosarcoma; LMP, latent membrane protein; CTLs, cytotoxic T-cell lymphocytes.
